# SPNeoDeath: A demographic and epidemiological dataset having infant, mother, prenatal care and childbirth data related to births and neonatal deaths in São Paulo city Brazil – 2012–2018

**DOI:** 10.1016/j.dib.2020.106093

**Published:** 2020-07-30

**Authors:** Carlos Eduardo Beluzo, Everton Silva, Luciana Correia Alves, Rodrigo Campos Bresan, Natália Martins Arruda, Ricardo Sovat, Tiago Carvalho

**Affiliations:** a Federal Institute of São Paulo, Campinas-SP, Brazil; b Department of Demography, University of Campinas (UNICAMP), Brazil

**Keywords:** Neonatal mortality, Newborn, Maternal, Birth, Death, Demographic, Epidemiological

## Abstract

**SPNeodeath** dataset includes births and deaths of infants during the neonatal period from São Paulo city between 2012 and 2018, containing more than 1.4 million records. The dataset was created from SINASC and SIM Brazilian information systems for births and deaths respectively. SINASC comprises information about demographic and epidemiological data for the infant, mother, prenatal care and childbirth. SIM collects information about mortality, and it is used as the basis for the calculation of vital statistics, such as neonatal mortality rate. SIM was only used to label records from SINASC, when the death happened until 28 days of life. **SPNeodeath** has 23 variables with socioeconomic maternal condition features, maternal obstetrics features, newborn related features and previous care related features, besides a label feature describing if the subject survived, or not, after 28 days of life. In order to build the dataset, DBF files were downloaded from DATASUS ftp repository and converted to CSV format, the R programming language, and then the CSV files were processed using Python programming language. Features with incorrect values and unknowing information were removed.

**Specifications Table**

**Table:** 

**Subject**	Public Health and Health Policy
**Specific subject area**	Demographic and epidemiological data for the infant, mother, prenatal care and childbirth of Births and Neonatal Deaths
**Type of data**	text/csv
**How data were acquired**	Official records of the national healthcare system.
**Data format**	Mixed (raw, analysed and filtered).
**Parameters for data collection**	Demographic and epidemiological data from the infant, mother, prenatal care and childbirth of Births and Neonatal Deaths from São Paulo city Brazil between the years of 2012 and 2018
**Description of data collection**	The data were extracted from SINASC and SIM. SINASC collects information from births that happened in all national territory, both in the public and private health sectors and in households and it is done in the municipal context. Its main instrument is the declaration of live births (DN) – right after the birth in the place where the birth occurred, a health professional, properly trained must fill all the fields in the DN. SIM collects death information and uses the death declaration form (DO). The data are collected in the health providers and in registry offices
**Data source location**	Health Informatics Department of the Brazilian Ministry of Health – DATASUS (ftp.datasus.gov.br)
**Data accessibility**	https://doi.org/10.7303/syn22240254
**Related research article**	C.E. Beluzo, E. Silva, L.C. Alves, R.C. Bresan, N.M. Arruda, R.B. Sovat, T. Carvalho, Towards Neonatal Mortality Risk Classification: a data-driven approach using neonatal, maternal, and social factors Informatics in Medicine Unlocked, Elsevier. In Press.

**Value of the Data**

• SPNeoDeath is a dataset that provides more than 1.4 million samples representing births and deaths in the city of São Paulo-Brazil between 2012 and 2018.
• Dataset intends to support research focused in understanding neonatal mortality (NM) and its associated factors, providing a set of 24 features associated with NM, divided in 3 main groups: (1) socioeconomic maternal conditions features, (2) maternal obstetrics features and, (3) features related to the newborn.
• Research from fields as medicine, demography, public health, and computer science are some of the main groups which can be beneficiated by the proposed dataset.
• Researchers interested on how specific factors relate to NM evolution along years (causal studies along years), or interested in simulating the impact of variability across specific factors over NMR are just some examples of uses for this dataset.

**Table 1:** SPNeoDeath dataset data dictionary.

***Variable name***	***Description***	***Data domain***
*Demographic and socioeconomic variables*		
**maternal_age**	Mother's age	Quantitative Continuous (integer) [10..63]
**tp_maternal_skin_color**	Mother's race/skin color	Categorical Nominal (integer) 1 – White; 2 – Black; 3 – Yellow; 4 – Brown Skin; 5 – Indigenous.
**tp_marital_status**	Mother's marital status	Categorical Nominal (integer) 1 – Single; 2 – Married; 3 – Widow; 4 – Judicially separated/divorced; 5 – Common-law marriage; 9 – Ignored.
**tp_maternal_education_years**	Mother's years of schooling	Categorical Nominal (integer) 1 – None; 2 – from 1 to 3 years; 3 – from 4 to 7 years; 4 – from 8 to 11 years; 5 – 12 and more; 9 – Ignored.
*Maternal obstetrics variables*		
**num_live_births**	Number of live births	Quantitative Continuous (integer) [0..18]
**num_fetal_losses**	Number of fetal losses	Quantitative Continuous (integer) [0..19]
**num_gestations**	Number of previous gestations	Quantitative Continuous (integer) [0..39]
**num_normal_labors**	Number of normal deliveries (labors)	Quantitative Continuous (integer) [0..41]
**num_cesarean_labors**	Number of cesarean deliveries (labors)	Quantitative Continuous (integer) [0..41]
**tp_pregnancy**	Type of pregnancy	Categorical Nominal (integer) 1 – Singleton; 2 – Twin; 3 – Triplet or more; 9 – Ignored.
*Previous care related variables*		
**tp_labor**	Child-birth type (labor type)	Categorical Nominal (integer) 1 – Vaginal; 2 – Cesarean;
**tp_prenatal_appointments**	Number of prenatal appointments by ranges	Categorical Ordinal (integer) 1 – None; 2 – from 1 to 3; 3 – from 4 to 6; 4 – 7 and more; 9 – Ignored.
**cd_robson_group**	Robson group classification	Categorical Ordinal (integer) [1..10]
*Newborn related variables*		
**tp_presentation_newborn**	Newborn presentation type	Categorical Nominal (integer) 1 – Cephalic; 2 – Pelvic or breech; 3 – Transverse; 9 – Ignored.
**has_congenital_malformation**	Presence of congenital malformation	Categorical Nominal (integer) 1 – Yes; 2 – No; 9 - Ignored
**newborn_weight**	Birth weight in grams	Quantitative Continuous (integer) [0..9999]
**cd_apgar1**	1-minute Apgar score	Categorical Ordinal (integer)[0..10]
**cd_apgar5**	5-min Apgar score	Categorical Ordinal (integer) [0..10]
**num_gestational_weeks**	Week of gestation (by ranges)	Categorical Ordinal (integer) [15..46]
**tp_birth_place**	Birth place	Categorical Nominal (integer) 1 – Hospital; 2 – Other health facilities; 3 – Home birth; 4 – Others.
**tp_childbirth_assistance**	Childbirth care	Categorical Nominal (integer) 1 – Doctor; 2 – nurse or obstetrician; 3 – Midwife; 4 – others; 9 – Ignored.
**p_fill_form_responsible**	Main worker role	Categorical Nominal (integer) 1 – Doctor; 2 – Nurse; 3 – Midwife; 4 – Registry Office employee; 5 – Others.
**tp_pregnancy_duration**	Gestational weeks by ranges	Categorical Ordinal (integer) 1 –Less than 22 weeks; 2 – 22 to 27 weeks; 3 – 28 to 31 weeks; 4 – 32 to 36 weeks; 5 – 37 to 41 weeks; 6 – 42 weeks and more; 9 – Ignored
**neonatal_death**	Death before 28 days (label)	Categorical Nominal (integer) 0 – survivor; 1 – dead.

## Data description

SPNeodeath dataset is based on secondary data of births and deaths of infants (from neonatal period only, i.e., when the child died within the first 28 days of life) from the city of São Paulo – Brazil between 2012 and 2018, comprising 1,427,906 rows and 24 columns. The data came from Mortality Information System (SIM - Sistema de Informação de Mortalidade) and the National Information System on Live Births (SINASC – Sistema de Informação de Nascidos Vivos), both from DATASUS (Health Informatics Department of the Brazilian Ministry of Health).

SINASC is fed using the Live Birth Statement (DNV – Declaração de Nascido Vivo) [1]. It comprises information about demographic and epidemiological data from the infant, mother, prenatal care and childbirth. Similarly, we have the Death Certificate (DO – Declaração de Óbito) that is the document used to collect information about mortality and it is used as the basis for the calculation of vital statistics, such as the calculation of the Brazilian neonatal mortality rate. SIM has the main goal of supporting the collection, storage and management process of death records in Brazil [2], and was used to label records from SINASC, where death happened until 28 days of life, by using DNV as an association key, since it is a common field in both systems.

Each sample in our final dataset comprises some features from SINASC, and a label feature describing if the subject survived, or not, after 28 days of life. The other 23 features can be categorized in four groups: (a) socioeconomic maternal conditions features: includes features such as mother's age, years of schooling, marital status and race/skin color; (b) maternal obstetrics features: number of live births, number of previous fetal losses, number of previous pregnancies, number of normal and caesarean labors and type of pregnancy; (c) newborn related features: birth weight, number of pregnancy weeks, Apgar score at 1st minute, Apgar score at 5th minute, congenital anomaly and type of presentation of the newborn; and (d) previous care related features: number of prenatal consultations, labor type, childbirth care and Robson 10-groups classification. A detailed description of features is shown in Table 1.

**Fig. 1:**
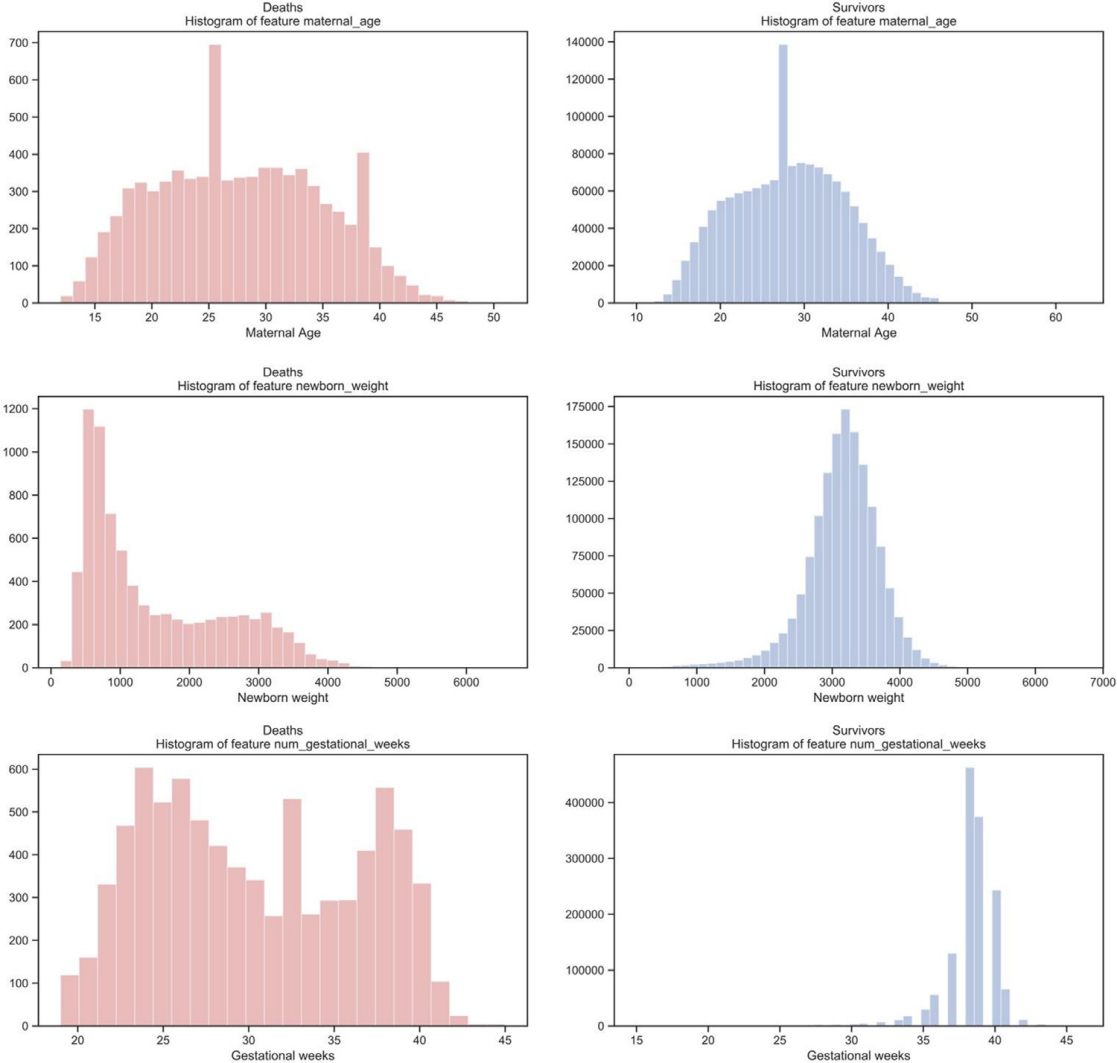
Histogram of quantitative features.

**Fig. 2:**
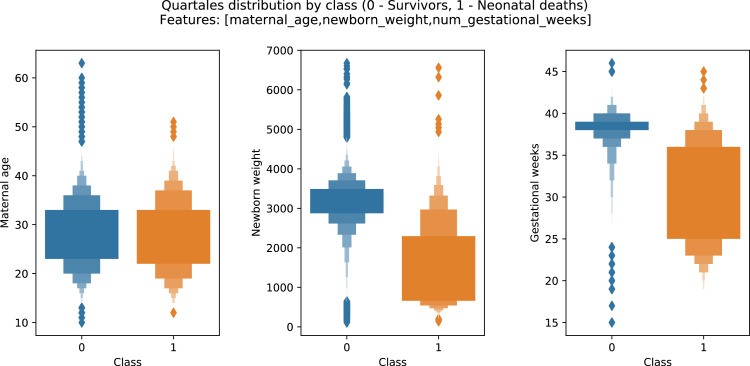
Boxplot quartiles of quantitative features.

**Fig. 3:**
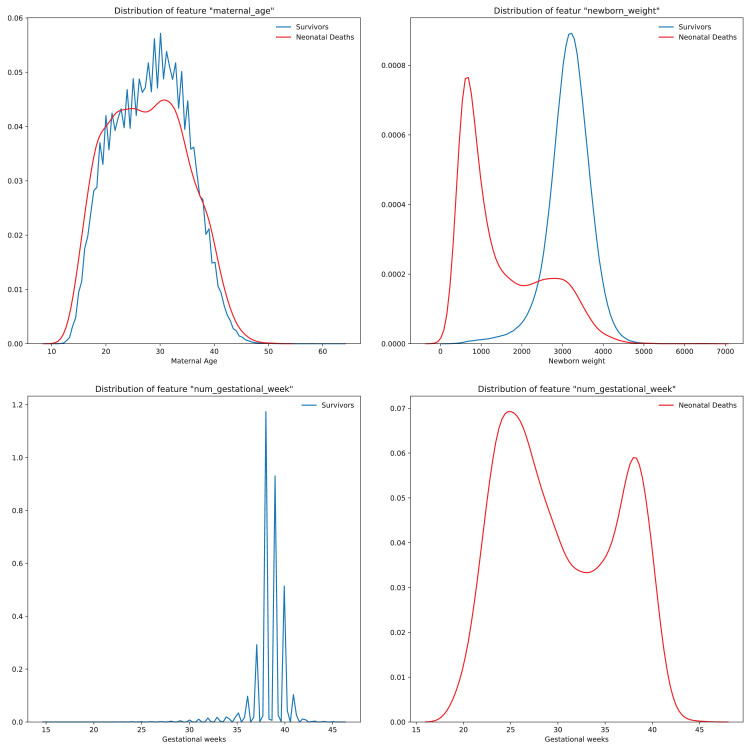
Quantitative features’ data distribution.

**Fig. 4:**
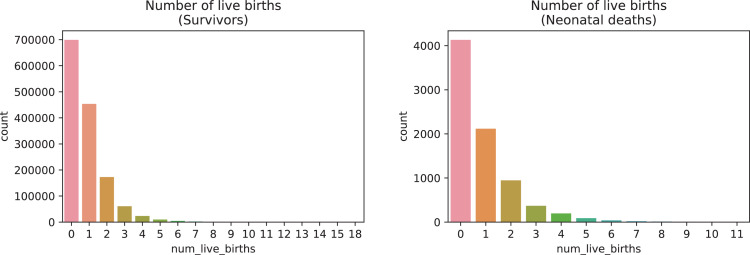
Value counts of feature *num_live_births*.

**Fig. 5:**
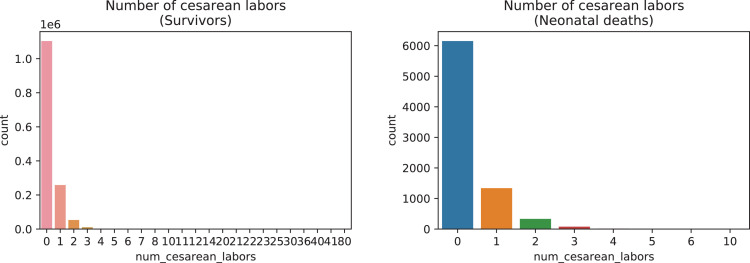
Value counts of feature *num_fetal_losses*.

**Fig. 6:**
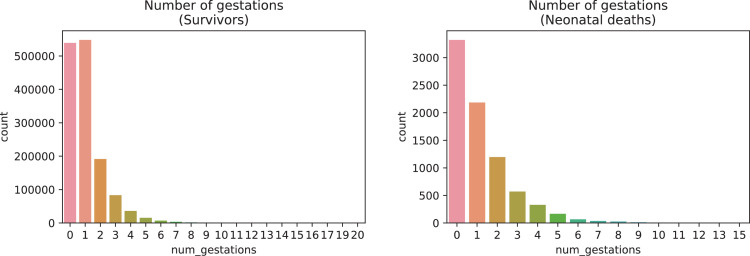
Value counts of feature *num_gestations*.

**Fig. 7:**
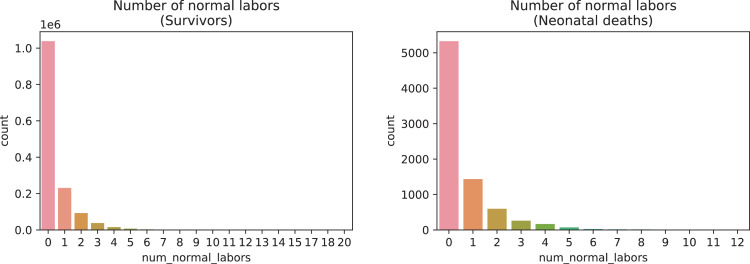
Value counts of feature *num_normal_labors.*

**Fig. 8:**
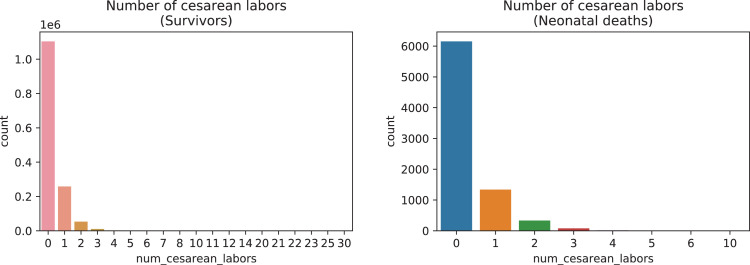
Value counts of feature *num_cesarean_labors.*

**Fig. 9:**
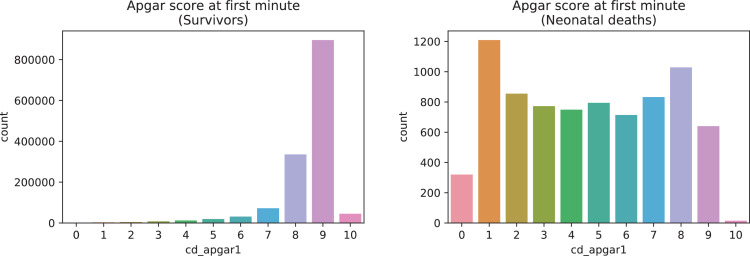
Value counts of feature *cd_apgar1*.

**Fig. 10:**
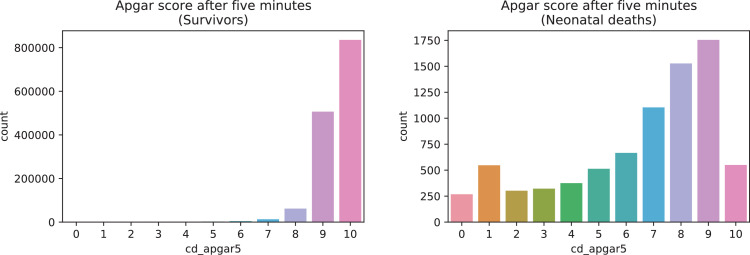
Value counts of feature *cd_agpar5*.

**Fig. 11:**
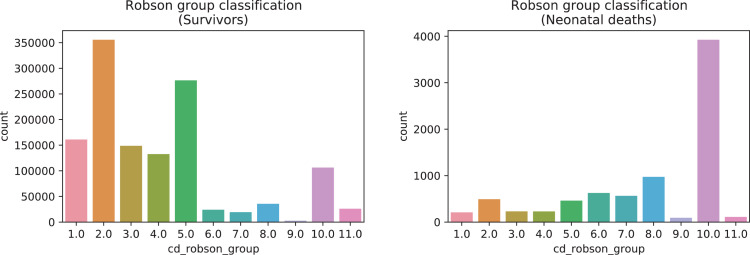
Value counts of feature *cd_robson_group*.

**Fig. 12:**
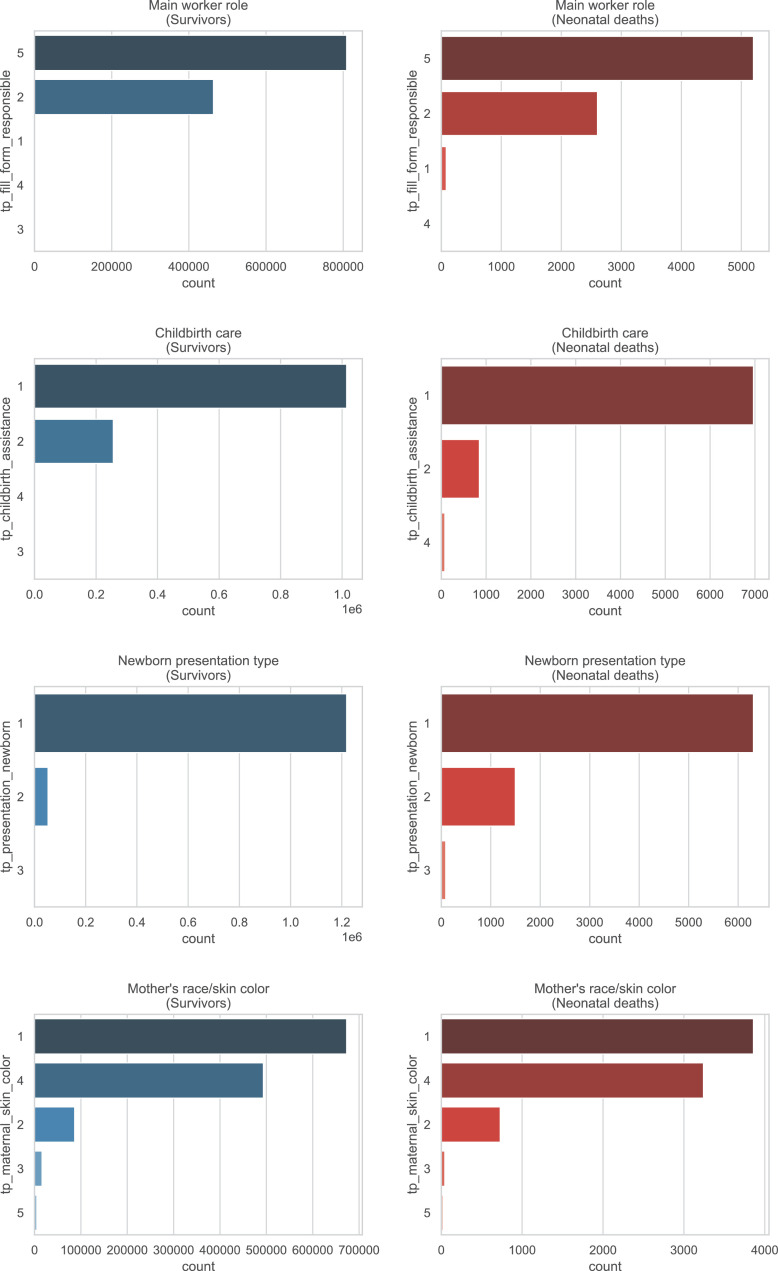
Value counts of categorical nominal features *tp_fill_form_responsible, tp_childbirth_assistance, tp_presentation_newborn and tp_maternal_skin_color* separated by survivor and death samples.

**Fig. 13:**
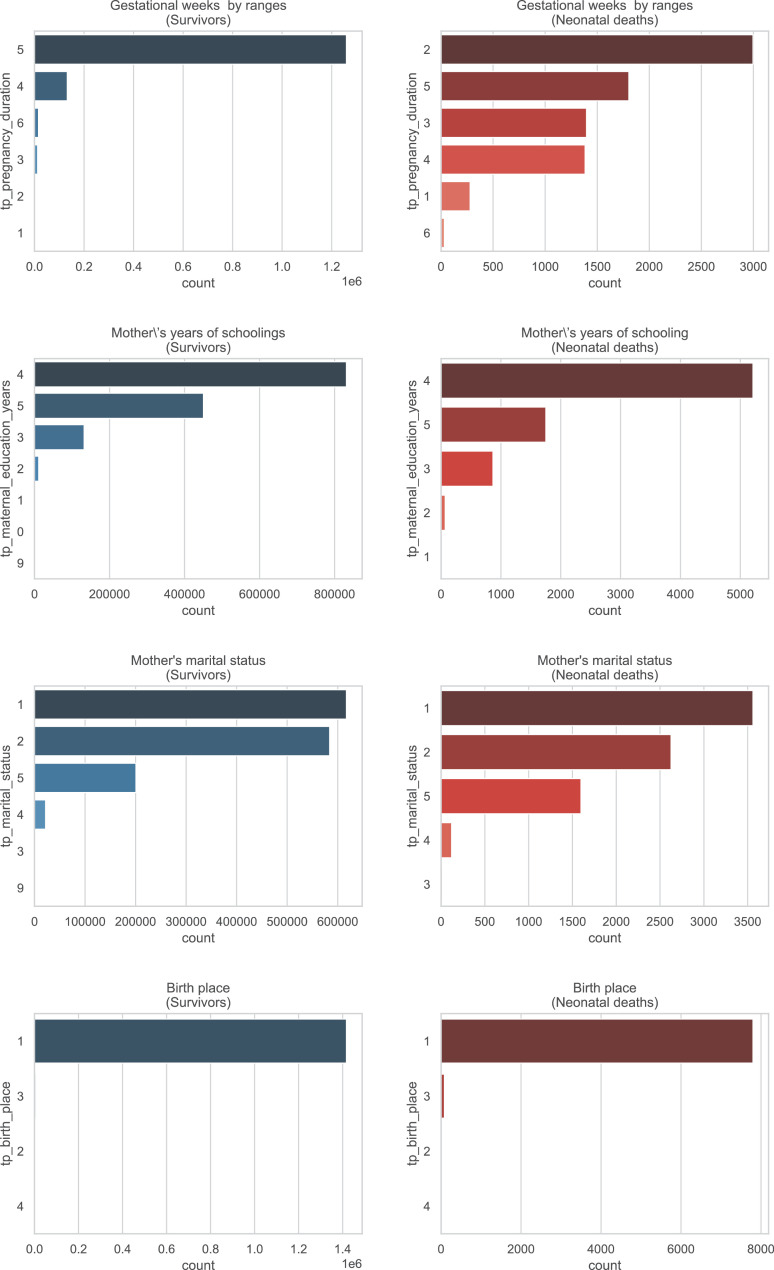
Values counts of categorical nominal features *has_congenital_malformation, tp_prenatal_appointments, tp_labor* and *tp_pregnancy* separated by survivor and death samples.

**Fig. 14:**
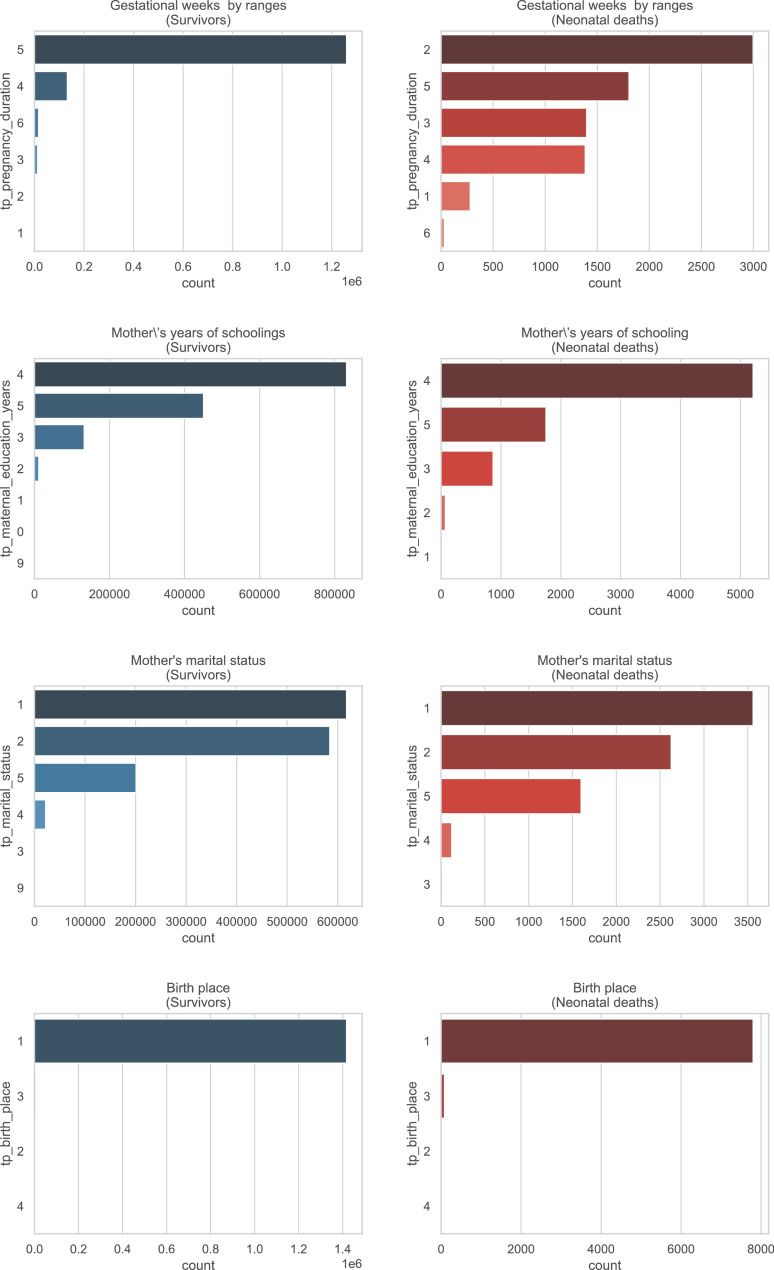
Values counts of categorical nominal feature *tp_pregnancy_duration, tp_maternal_education_years, tp_marital_status* and *tp_birth_place* separated by survivor and death samples.

A brief insight on dataset features values distribution is presented here using graphs. Considering that relevant differences can be observed between the two classes, survivors or neonatal death, the graphs show values separated by each of these classes. For the quantitative continuous features maternal age, newborn weight and gestational weeks, histograms are presented in Fig. 1, boxplot quartiles in Fig. 2 and data distribution in Fig. 3. For the categorical ordinal features, values count, and proportion are presented in Figs. 4**–**11**.** Also, for each, a descriptive table with counts and data values proportions are presented in Tables 2**–**9. Finally for categorical nominal features there is also values count bar plots on Figs. 12**–**14.

**Table 2:** Value counts and proportions of feature *num_live_births*.

Survivors			Deaths		
num_live_births	Count	Proportion (%)	num_live_births	Count	Proportion (%)
0	698,953	48.95	0	4130	52.09
1	453,503	31.76	1	2117	26.7
2	172,725	12.1	2	946	11.93
3	60,959	4.27	3	368	4.64
4	23,481	1.64	4	196	2.47
5	9815	0.69	5	90	1.14
6	4521	0.32	6	40	0.5
7	1987	0.14	7	19	0.24
8	983	0.07	8	12	0.15
9	484	0.03	9	6	0.08
10	308	0.02	11	3	0.04
11	107	0.01	10	1	0.01
12	43	0.0			
13	29	0.0			
14	6	0.0			
15	1	0.0			
18	1	0.0

**Table 3:** Value counts and proportions of feature *num_fetal_losses*.

Survivors			Deaths		
num_fetal_losses	Count	Proportion (%)	num_fetal_losses	Count	Proportion (%)
0	1,180,226	82.65	0	5946	75.0
1	198,744	13.92	1	1475	18.6
2	37,178	2.6	2	342	4.31
3	8470	0.59	3	100	1.26
4	2027	0.14	4	35	0.44
5	711	0.05	5	14	0.18
6	258	0.02	6	11	0.14
7	139	0.01	7	2	0.03
8	51	0.0	8	2	0.03
10	42	0.0	13	1	0.01
9	33	0.0			
11	15	0.0			
12	5	0.0			
13	2	0.0			
14	2	0.0			
18	1	0.0			
17	1	0.0			
19	1	0.0

**Table 4:** Value counts and proportions of feature *num_gestations*.

Survivors			Deaths		
num_gestations	Count	Proportion (%)	num_gestations	Count	Proportion (%)
1	548,117	38.39	0	3322	41.9
0	539,248	37.76	1	2186	27.57
2	191,556	13.42	2	1197	15.1
3	83,289	5.83	3	571	7.2
4	36,136	2.53	4	330	4.16
5	15,556	1.09	5	167	2.11
6	7094	0.5	6	67	0.85
7	3498	0.24	7	37	0.47
8	1644	0.12	8	26	0.33
9	884	0.06	9	13	0.16
10	420	0.03	10	6	0.08
11	229	0.02	15	2	0.03
12	112	0.01	11	2	0.03
13	56	0.0	13	1	0.01
14	35	0.0	12	1	0.01
16	8	0.0			
15	7	0.0			
19	4	0.0			
17	3	0.0			
39	3	0.0			
38	2	0.0			
[20, 21, 33, 27, 30]	5	0.0

**Table 5:** Value counts and proportions of feature *num_normal_labors.*

Survivors			Deaths		
num_normal_labors	Count	Proportion (%)	num_normal_labors	Count	Proportion (%)
0	1,037,754	72.68	0	5326	67.18
1	230,902	16.17	1	1434	18.09
2	92,837	6.5	2	599	7.56
3	37,415	2.62	3	262	3.3
4	15,721	1.1	4	166	2.09
5	6971	0.49	5	73	0.92
6	3290	0.23	6	26	0.33
7	1514	0.11	7	18	0.23
8	720	0.05	8	14	0.18
9	367	0.03	9	6	0.08
10	225	0.02	12	2	0.03
11	90	0.01	11	1	0.01
12	40	0.0	10	1	0.01
13	22	0.0			
20	12	0.0			
14	6	0.0			
22	3	0.0			
37	3	0.0			
23	2	0.0			
[15, 17, 18, 21, 25]	5	0.0			
[30, 32, 33]	3	0.0			
[38, 40, 41, 80]	4	0.0

**Table 6:** Value counts and proportions of feature *num_cesarean_labors.*

Survivors			Deaths		
num_cesarean_labors	Count	Proportion (%)	num_cesarean_labors	Count	Proportion (%)
0	1,103,570	77.29	0	6153	77.61
1	258,344	18.09	1	1338	16.88
2	52,959	3.71	2	333	4.2
3	10,433	0.73	3	77	0.97
4	2018	0.14	4	15	0.19
5	372	0.03	5	6	0.08
6	83	0.01	6	4	0.05
10	40	0.0	10	2	0.03
7	25	0.0			
11	22	0.0			
20	13	0.0			
30	4	0.0			
22	4	0.0			
8	4	0.0			
14	3	0.0			
25	2	0.0			
21	2	0.0			
40	2	0.0			
41	2	0.0			
80	1	0.0			
23	1	0.0			
36	1	0.0			
12	1	0.0

**Table 7:** Value counts and proportions of feature *cd_apgar1*.

Survivors			Deaths		
cd_apgar1	Count	Proportion (%)	cd_apgar1	Count	Proportion (%)
9	895,748	62.73	1	1209	15.25
8	335,711	23.51	8	1028	12.97
7	71,859	5.03	2	855	10.78
10	44,843	3.14	7	832	10.49
6	31,230	2.19	5	794	10.02
5	19,296	1.35	3	772	9.74
4	12,264	0.86	4	749	9.45
3	7882	0.55	6	714	9.01
2	4603	0.32	9	640	8.07
1	2920	0.2	0	320	4.04
0	1550	0.11	10	15	0.19

**Table 8:** Value counts and proportions of feature *cd_agpar5*.

Survivors			Deaths		
cd_apgar5	Count	Proportion (%)	cd_apgar5	Count	Proportion (%)
9	1754	22.12	10	835639	58.52
8	1527	19.26	9	506557	35.48
7	1104	13.93	8	61956	4.34
6	666	8.4	7	12803	0.9
10	550	6.94	6	4231	0.3
1	547	6.9	5	2395	0.17
5	513	6.47	4	1184	0.08
4	375	4.73	1	1086	0.08
3	322	4.06	0	936	0.07
2	302	3.81	2	567	0.04
0	268	3.38	3	552	0.04

**Table 9:** Value counts and proportions of feature *cd_robson_group.*

Survivors			Deaths		
cd_robson_group	Count	Proportion (%)	cd_robson_group	Count	Proportion (%)
2.0	355,673	27.6	10.0	3926	49.6
5.0	276,398	21.45	8.0	974	12.31
1.0	161,125	12.5	6.0	626	7.91
3.0	148,799	11.55	7.0	566	7.15
4.0	132,573	10.29	2.0	492	6.22
10.0	106,226	8.24	5.0	462	5.84
8.0	35,722	2.77	3.0	230	2.91
11.0	26,066	2.02	4.0	229	2.89
6.0	24,042	1.87	1.0	209	2.64
7.0	19,221	1.49	11.0	110	1.39
9.0	2640	0.2	9.0	91	1.15

## Experimental design, materials and methods

The raw data from SINASC and SIM can be obtained directly from DATAUS website. Originally, the files are on DBF format, a standard database file used by dBASE database management system. In order to read the DBF files and convert then to CSV format, a library from R programming language was used. Then the CSV files were loaded into a development environment using Python programming language, and by using Pandas library, all data manipulation was performed. The **SPNeoDeath** dataset is available in CSV format.

SINASC and SIM datasets are not initially linked, so to associate birth and death records, a simple combination between the datasets was performed using a common variable for both systems, Number of Live Birth Statement (NUMERODN). Even though filling out the DNV and the DO is mandatory, there is a significant deficiency in data quality due to many situations such as loss when sending the data from hospitals to the city health offices, fields filled with incorrect values and unknown information by the person answering.

After the combination, a new field was added in the resultant data set to label the samples as being a neonatal death (deaths occurred before the first 28 days of life) or not. This was achieved by calculating the difference between the birth date (from SINASC) and the death date (from SIM).

SIM data are applied just for labelling purposes, so for each SINASC record, SIM data were used to label the sample as dead or alive class, makingit possible to construct a big annotated dataset. After the linkage between SIM and SINASC, the key used on the joining operation was removed from the resultant dataset, as well as many other fields that could be used to re-identify individuals. As SIM data is used just to allow data set labelling, after this process all SIM fields were also removed from the final dataset.

As mentioned, in the context of Brazilian public health data, occurrence of missing or inconsistent data is common and it mostly happens due to the incorrect filling of handwritten forms. Rows having fields with inconsistent values were removed, and a general approach for demographic studies to deal with missing values were used based on approaches of similar studies [[3], [4], [5]]. All the features had less than 12% of missing values and basically, two different techniques were applied: for non-categorical features, with continuous numerical values, the mean value for this feature in the dataset is calculated, and the feature is filled with this value; whereas features with categorical values (discrete values) are filled using the most frequent value for this feature in the dataset (the mode value).

## Ethics statement

This paper uses publicly available data (SIM and SINASC) that has been de-identified and was deemed exempt from approval from a human research ethics committee.

## Acknowledgment

This research was supported by 10.13039/100000865Bill & Melinda Gates Foundation (Process no: OPP1201970) and 10.13039/501100006506Ministry of Health of Brazil, through the National Council for Scientific and Technological Development (CNPq) (Process no: 443774/2018-8). It was also supported by NVIDIA, that donated a GPU XP Titan used by the research team.

## Declaration of Competing Interest

The authors declare that they have no known competing financial interests or personal relationships which have, or could be perceived to have, influenced the work reported in this article.
